# Characterization of CIPK Family in Asian Pear (*Pyrus bretschneideri* Rehd) and Co-expression Analysis Related to Salt and Osmotic Stress Responses

**DOI:** 10.3389/fpls.2016.01361

**Published:** 2016-09-07

**Authors:** Jun Tang, Jing Lin, Hui Li, Xiaogang Li, Qingsong Yang, Zong-Ming Cheng, Youhong Chang

**Affiliations:** ^1^Jiangsu Key Laboratory for Horticultural Crop Genetic Improvement, Institute of Horticulture, Jiangsu Academy of Agricultural SciencesNanjing, China; ^2^Department of Plant Sciences, University of Tennessee at Knoxville, KnoxvilleTN, USA

**Keywords:** Asian pear, CIPK, co-expression, evolution, osmotic stress, salt stress

## Abstract

Asian pear (*Pyrus bretschneideri*) is one of the most important fruit crops in the world, and its growth and productivity are frequently affected by abiotic stresses. Calcineurin B-like interacting protein kinases (CIPKs) as caladium-sensor protein kinases interact with Ca^2+^-binding CBLs to extensively mediate abiotic stress responses in plants. Although the pear genome sequence has been released, little information is available about the *CIPK* genes in pear, especially in response to salt and osmotic stresses. In this study, we systematically identified 28 *CIPK* family members from the sequenced pear genome and analyzed their organization, phylogeny, gene structure, protein motif, and synteny duplication divergences. Most duplicated *PbCIPK*s underwent purifying selection, and their evolutionary divergences accompanied with the pear whole genome duplication. We also investigated stress -responsive expression patterns and co-expression networks of CIPK family under salt and osmotic stresses, and the distribution of stress-related *cis*-regulatory elements in promoter regions. Our results suggest that most *PbCIPK*s could play important roles in the abiotic stress responses. Some *PbCIPK*s, such as *PbCIPK22*, *-19*, *-18*, *-15*, *-8*, and *-6* can serve as core regulators in response to salt and osmotic stresses based on co-expression networks of *PbCIPK*s. Some sets of genes that were involved in response to salt did not overlap with those in response to osmotic responses, suggesting the sub-functionalization of *CIPK* genes in stress responses. This study revealed some candidate genes that play roles in early responses to salt and osmotic stress for further characterization of abiotic stress responses medicated by *CIPK*s in pear.

## Introduction

Plants often encounter abiotic stresses, such as high salinity, drought, and low temperatures, which can adversely affect their growth, development, and productivity during their life cycles. To cope with these stresses, plants have evolved complex strategies to perceive, transduce, and respond to stresses at the molecular, cellular, and physiological levels (Zhu, 2002; Yamaguchi-Shinozaki and Shinozaki, 2006; Manik et al., 2015). During response and adaptation to stresses, calcineurin B-like (CBL) interacting protein kinases (CIPKs) have been reported to be involved in the stress responses (Kim et al., 2003; Kolukisaoglu et al., 2004). CIPK proteins, also known as SnRK3 proteins, are an important category of serine/threonine protein kinases in plants. The CIPK genes consist of an N-terminal protein kinase domain similar to those found in other plant protein kinases and a unique C-terminal regulatory domain (Luan, 2009; Chaves-Sanjuan et al., 2014; Kleist et al., 2014). The C-terminal regulatory domain of CIPKs contain the conserved NAF domain (Pfam no. PF03822) that acts as a self-inhibitory motif by interacting with Ca^2+^-binding CBLs to activate the kinase catalytic activity of CIPK, which decodes calcium signals elicited during stress stimuli (Albrecht et al., 2001; Weinl and Kudla, 2009; Chaves-Sanjuan et al., 2014; Pandey et al., 2015).

Calcineurin B-like interacting protein kinases are extensively involve in plant stress responses. The salt overly sensitive (SOS) pathway was the first identified CBL-CIPK pathway for maintaining ion homeostasis in plant cells (Qiu et al., 2002). The SOS pathway contains three components, SOS3 (a Ca^2+^ sensor of the CBL family), SOS2 (a CIPK protein), and SOS1 (a Na^+^/H^+^ antiporter activated by SOS2), that have been well characterized for their roles in maintaining ion homeostasis during salt stress (Xiong et al., 2002; Qiu et al., 2004; Ji et al., 2013). Overexpression of single or common SOS pathway genes can enhance salt tolerance in transgenic plants (Qiu et al., 2002; Guo et al., 2004; Batelli et al., 2007; Ma et al., 2014). Additionally, the SOS pathway, in response to salinity stress, is also functionally conserved in plants (Martinez-Atienza et al., 2007; Tang et al., 2010). Moreover, some *CIPK* genes in Rosaceae species function similarly to *AtCIPK24*/*AtSOS2* in salt tolerance (Hu et al., 2012; Wang R.K. et al., 2012).

Asian pear (*Pyrus bretschneideri*) is one of the most important fruit crops in the family of *Rosaceae*, and is widely cultivated in world. Pear is frequently affected by abiotic stress, such as salinity, drought or osmotic stress, which affect growth, development, and productivity. Although the pear genome sequence has been released (Wu et al., 2013), up to now, the CIPK family has not been fully characterized specifically. How *CIPK* genes respond to salt or osmotic stress is still unclear. In this study, we performed a genome-wide identification and analysis of CIPK family in pear, and 28 *PbCIPK* genes were analyzed for family organization, gene structure and conserved motif, genomic location, gene duplication, evolutionary divergence, stress-related *cis*-elements, and expression patterns, and co-expression networks under salt and osmotic stresses. Systematic analyses indicated that most duplicated *PbCIPK*s underwent the purifying selection and evolutionary divergences accompanied with pear whole genome duplications. Moreover, stress-responsive *cis*-element analysis revealed the possible roles of *PbCIPK* genes to respond to abiotic stresses. Expression analysis of *PbCIPK*s under salt and osmotic stresses indicated that some *CIPK*s are involved in the co-response of salt and osmotic stresses, whereas others were sub-functionalized in response to salt stress and osmotic stress, respectively. Additionally, based on gene co-expression analysis of *PbCIPK*s, we identified *PbCIPK22*, *-19*, *-18*, *-15*, *-8*, and *-6* as core regulators in response to salt and osmotic stresses. This work indicates the roles of *CIPK* genes in pear in response to abiotic stress and provides some basic information for stress-resistance studies of *CIPK*s.

## Materials and Methods

### Database Searches and Identification of CIPK Genes in Asian Pear

Whole genome annotation sequences of Asian pear (*P. bretschneideri*) were collected from the pear genome project^1^ (Wu et al., 2013). The CIPK sequences of the *Arabidopsis* (Hrabak et al., 2003) and rice (Xiang et al., 2007) were downloaded from The Arabidopsis Information Resource (TAIR)^2^ and Rice Genome Annotation Project (RGAP)^3^, respectively. The hidden Markov model based profiles (HMM-profiles) of Pkinase (PF00069) and NAF (PF03106) were downloaded from the Pfam database^4^ (Finn et al., 2014), and used to search against all proteins sequences of pear using HMMER3.0^5^. In addition, all retrieved CIPKs from *Arabidopsis* as queries were used to search against pear proteome by local BLAST (McGinnis and Madden, 2004) with identity >50% according to the previously described (Jimenez et al., 2015). The results of the combined searches, and all non-redundant sequences were subjected to the Pfam^ 4^ and SMART^6^ database for domain analysis. The sequences were only accepted if they simultaneously contained the conserved Pkinase domain and the NAF domain as putative *PbCIPK* gene models. As a result, 28 *PbCIPK* family genes were identified and analyzed from pear genome, and the genes were named according to their corresponding genomic locations. Another five CIPKs were located in three scaffolds, but they were considered incomplete due to lack of chromosomal information (**Table 1**).

**Table 1:** The characteristics of CIPK family members in pear (*P. bretschneideri*).

Gene name	Gene ID	Position	No. of Intron	CDS (bp)	Size (aa)	MW	pI	Type of domain	*Arabidopsis* ortholog
PbCIPK1	Pbr007816.1	Chr1:1823077..1826756(-)	14	1284	427	48.74	8.25	Pkinase, NAF	CIPK9
PbCIPK2	Pbr003161.1	Chr2:21926798..21931377(+)	13	1341	446	50.68	8.59	Pkinase, NAF	CIPK24/SOS2
PbCIPK3	Pbr013115.1	Chr3:22431966..22433979(+)	0	1389	462	51.60	8.75	Pkinase, NAF	CIPK11/SIP4
PbCIPK4	Pbr013072.1	Chr3:22763880..22766497(+)	0	1371	456	52.08	8.92	Pkinase, NAF	CIPK10
PbCIPK5	Pbr000505.2	Chr5:24945487..24947705(+)	0	789	262	29.47	4.84	Pkinase, NAF	CIPK12
PbCIPK6	Pbr000504.1	Chr5:24951909..24954070(-)	0	1392	463	52.03	9.20	Pkinase, NAF	CIPK20
PbCIPK7	Pbr011665.1	Chr6:18116683..18123297(+)	11	1368	455	50.89	6.31	Pkinase, NAF	CIPK1
PbCIPK8	Pbr019032.1	Chr8:14318721..14320721(-)	0	1323	440	49.59	8.62	Pkinase, NAF	CIPK25
PbCIPK9	Pbr016120.1	Chr10:3521730..3523693(+)	0	1491	496	55.33	8.15	Pkinase, NAF	CIPK12
PbCIPK10	Pbr016121.1	Chr10:3528604..3529995(-)	0	1392	463	51.75	8.97	Pkinase, NAF	CIPK20
PbCIPK11	Pbr042907.1	Chr10:6223214..6227972(-)	14	1416	471	52.47	9.50	Pkinase, NAF	CIPK23
PbCIPK12	Pbr011565.1	Chr11:25408733..25411205(+)	0	1410	469	53.66	8.83	Pkinase, NAF	CIPK10
PbCIPK13	Pbr035731.1	Chr11:25967788..25970002(+)	0	1167	388	43.44	6.89	Pkinase, NAF	CIPK11/SIP4
PbCIPK14	Pbr014616.1	Chr12:4644525..4645865(+)	0	1341	446	50.18	7.26	Pkinase, NAF	CIPK14
PbCIPK15	Pbr014612.1	Chr12:4660284..4661735(-)	0	1452	483	54.64	8.98	Pkinase, NAF	CIPK10
PbCIPK16	Pbr014711.1	Chr13:5124406..5126030(+)	0	1320	439	48.22	9.70	Pkinase, NAF	CIPK7
PbCIPK17	Pbr018565.1	Chr13:7679861..7690736(+)	10	1233	410	45.86	8.22	Pkinase, NAF	CIPK1
PbCIPK18	Pbr020002.1	Chr15:5458702..5461003(+)	0	1335	444	50.22	8.83	Pkinase, NAF	CIPK25
PbCIPK19	Pbr034256.1	Chr15:11682777..11685059(+)	0	1167	388	43.77	9.21	Pkinase, NAF	CIPK6/SIP3
PbCIPK20	Pbr010647.1	Chr15:12835426..12839862(+)	13	1338	445	50.53	9.13	Pkinase, NAF	CIPK24/SOS2
PbCIPK21	Pbr007491.1	Chr15:38413957..38419417(-)	13	1350	449	51.09	7.67	Pkinase, NAF	CIPK8
PbCIPK22	Pbr037774.1	Chr16:5555693..5562205(+)	11	1368	455	50.91	6.31	Pkinase, NAF	CIPK1
PbCIPK23	Pbr022407.1	Chr17:3519219..3521163(+)	0	1392	463	52.56	9.42	Pkinase, NAF	CIPK25
PbCIPK24	Pbr015971.1	scaffold235.0:526619..529248(+)	0	1476	491	55.47	8.52	Pkinase, NAF	CIPK10
PbCIPK25	Pbr015975.1	scaffold235.0:579859..581518(-)	0	1308	435	49.15	7.99	Pkinase, NAF	CIPK11/SIP4
PbCIPK26	Pbr041843.1	scaffold958.0.1:41764..47451(+)	12	1206	401	45.75	6.27	Pkinase, NAF	CIPK3
PbCIPK27	Pbr041844.1	scaffold958.0.1:100240..105927(-)	12	1206	401	45.75	6.27	Pkinase, NAF	CIPK3
PbCIPK28	Pbr003572.1	scaffold1158.0:87853..89701(+)	0	1389	462	50.99	9.36	Pkinase, NAF	CIPK4


*bp, base pair; aa, amino acids; CDS, coding sequence; MW, molecular weight; pI, Isoelectric point.*

### Protein Properties and Sequence Analyses

Protein properties, including molecular weight (MW) and isoelectric point (pI) of PbCIPK, were predicted using the online tool Compute pI/Mw^7^. The motif analyses of PbCIPKs were detected using MEME software^8^ with default parameter settings, except the width of motifs was set from 6 to 60, the maximum number of motifs was 15. Identified motifs were annotated by the PROSITE^9^. Gene structure was analyzed using the GSDS 2.0^10^ based on alignments of genomic and CDS sequences of *PbCIPK*s that were retrieved from the pear genome database.

### Multiple Sequences Alignment and Phylogenetic Analysis

Multiple sequence alignments of CIPK proteins from pear, *Arabidopsis*, rice, and other species were performed using ClustalW 2.0 (Larkin et al., 2007). Phylogenetic trees were constructed using neighbor-joining (NJ) and maximum-likelihood (ML) method with bootstrap values 1000 replicates in MEGA 6.0 (Tamura et al., 2013). Data for CIPK proteins from *Brachypodium distachyon*, soybean (*Glycine max*), peach (*Prunus persica*), poplar (*Populus trichocarpa*), grape (*Vitis vinifera)* were obtained from Kleist et al. (2014).

### Chromosomal Locations and Synteny Analyses

To locate *PbCIPK* genes on chromosomes, the information of chromosomal position of each *PbCIPK* gene was obtained from the pear genome, and then used to construct a physical map by an in-house Perl script. Synteny analysis was carried out using the method described by (Tang et al., 2014). Briefly, BLASTP was performed to search for potential homologous gene pairs (*E*-value < 1*e* - 20, top five matches) in all protein sequences of pear. Then, the blast hits and gene locations were used as the inputs for MCScanX (Wang Y. et al., 2012) to investigate all potential paralogous duplication events in *P. bretschneideri* with the default settings. The paralogous duplicated pairs of *PbCIPK* family (**Table 2**) were identified and extracted and the synteny relationships of *PbCIPK* duplicated pairs shown using Circos (Krzywinski et al., 2009).

**Table 2:** Colinearity gene pairs and evolutionary selection in the pear CIPK family.

Duplicated Gene 1	Duplicated Gene 2	Duplicated type	Ks	Ka	Ka/Ks	MYA	Purifying selection
PbCIPK2	PbCIPK20	Segmental duplication	0.182	0.049	0.272	9.82	Yes
PbCIPK3	PbCIPK13	Segmental duplication	0.286	0.031	0.108	15.43	Yes
PbCIPK3	PbCIPK14	Segmental duplication	1.031	0.337	0.327	55.67	Yes
PbCIPK5	PbCIPK9	Segmental duplication	0.270	0.031	0.116	14.56	Yes
PbCIPK7	PbCIPK22	Segmental duplication	0.007	0.001	0.142	0.36	Yes
PbCIPK8	PbCIPK18	Segmental duplication	0.178	0.047	0.266	9.62	Yes
PbCIPK12	PbCIPK4	Segmental duplication	0.200	0.040	0.199	10.81	Yes
PbCIPK18	PbCIPK19	Segmental duplication	1.233	0.360	0.292	66.57	Yes
PbCIPK22	PbCIPK17	Segmental duplication	0.196	0.040	0.205	10.59	Yes


*Ka, non-synonymous substitution rate; Ks, synonymous substitution rate; MYA, Million years ago.*

### Selection Modes and *K*s Calculation

The ratio of non-synonymous substitutions (*K*a)/synonymous substitutions (*K*s) were evaluated to detect the modes of selection of *PbCIPK* genes. Coding sequences without stop codon of *PbCIPK* genes were aligned using ClustalW 2.0 (Larkin et al., 2007). Subsequently, *K*a, *K*s, and the ratio of *K*a/*K*s of the *PbCIPK* duplicated gene pairs were calculated using MEGA 6.0 (Tamura et al., 2013). The approximate divergence time (T) of the *PbCIPK* duplicated gene pairs were calculated based on the formula T = *K*s/2λ assuming clock-like rate (λ) of 9.26 synonymous substitutions per 10^9^ years (Lynch and Conery, 2000; Wu et al., 2013).

### *Cis*-Regulatory Elements in the Promoters of *PbCIPK* Genes

Based on the preliminary analysis of promoter region up to 2500 bp, we chose to analyze the -1000bp region due to concentrated stress-related *cis*-elements in the region and the upstream region greater than 1000 bp showed greater diversity with less consensus *cis*-elements. The 1000 bp upstream genomic sequences of *PbCIPK*s collected from the pear genome were analyzed for stress-responsive *cis*-acting elements, including ABRE (involved in the abscisic acid responsiveness), CE3 (involved in ABA and VP1 responsiveness), HSE (involved in heat and oxidative stress responsiveness), LTR (involved in the low-temperature responsiveness), MBR (MYB binding site involved in ABA and drought responsiveness), W-box (WRKY binding site involved in abiotic stress and defense response), and WUN-motif (involved in the wound responsiveness) in the promoters regions using PlantCARE^11^ and PLACE^12^ databases. The stress-related *cis*-elements in the 1000 bp upstream regions of *PbCIPK*s were collected and visualized by an in-house Perl script since our preliminary analysis showed most *cis*-elements were located in 1000 bp upstream region (data not shown).

### Plant Materials, Growth Conditions, and Stress Treatments

One-year-old shoots with young leaves from adult pear tree ‘Dangshansuli’ collected from the national germplasm orchard of the Institute of Horticulture, Jiangsu Academy of Agricultural Sciences (Nanjing, China, 32°02′16.2″N 118°52′16.1″E), were placed in the hydroponic containers containing 1/2 MS (Murashige and Skoog, 1962) solution (pH 5.8) and incubated in an artificial growth chamber at 22 ± 2°C with a photoperiod of 16-h light and 8-h dark, 60–70% humidity daily cycle. After 14 days, the robust shoots with young leaves were used for the experiments. For salt and osmotic treatments, shoots with young leaves of six individuals were treated using 1/2 MS solution (pH 5.8) containing 200 mM NaCl or 15% (w/v) polyethylene glycol (PEG6000). Control shoots were placed under the same growth conditions, but grown in 1/2 MS solution (pH 5.8) only. Young leaves were sampled according to (Li et al., 2013) under series treated time courses (0, 12, 24, and 48 h) in three biological replicates, flash frozen in liquid nitrogen, and stored at -80°C for RNA preparation.

### Real-Time Quantitative PCR (RT-qPCR)

Total RNA was isolated from samples using the RNAiso reagent (TaKaRa, Dalian, China) according to the manufacturer’s instructions. cDNA fragments were synthesized from total RNA using the TransScript^TM^ One-step gDNA Removal and cDNA Synthesis SuperMix (TaKaRa). RT-qPCR was performed using 7500 Fast Real-Time PCR System (Applied Biosystems, Carlsbad, CA, USA) instrument as described by (Tang et al., 2013). Each 20 μL amplification reaction contained 10 μL of SYBR premix Ex Taq^TM^ II (TaKaRa), 0.4 μL of each primer (10 μM), 7.2 μL of sterile, distilled water, and 2 μL of cDNA template. The PCR reaction contained an initial denaturation (95°C/2 min) followed by 40 cycles of 95°C denaturation for 10 s, 58°C annealing for 20 s and 72°C extension for 10 s. Gene specific primers were designed according to non-conserved region sequences of each *PbCIPK* gene using the program Beacon Designer 8.10^13^, and then the hit primer pairs of each gene were subjected to realign with the coding sequences of the whole pear genome using BLASTn. Finally, the primer pair was collected for RT-qPCR only when they both matched the same *PbCIPK* gene. Gene-specific primers are listed in Supplementary Table S1 and three technical replicates for each biological replicate were carried out. *PbACT2/7* and *PbUBQ10* genes of pear were used as internal controls for normalization according to Xu et al. (2015). The relative expression of *PbCIPK*s under salt and osmotic stress were calculated via the 2^-ΔΔCT^ method (Livak and Schmittgen, 2001).

### Co-expression Network Analysis

The topological relationships of stress responsive *PbCIPK* genes, a co-expression network analysis of *PbCIPK*s was performed and displayed based on Pearson correlation coefficient (PCC) of *PbCIPK*s’ expressions under salt and osmotic stresses. The PCC of *PbCIPK* gene pairs were calculated following the method of Tang et al. (2013). All of the gene-pairs of *PbCIPK*s with PCC at 0.05 significant level (*p*-value) were collected, and used to construct the co-expression networks using Cytoscape 3.3^14^. The nodes represent genes and the edges between nodes represent co-expression correlations of gene pairs. The different edge line types indicate different correlation levels, which designate different interaction strengths of co-regulated gene pairs. In addition, we compared protein physical interactional relationships of PbCIPK orthologs in *Arabidopsis* using STRING^15^ with the default program parameter settings.

## Results and Discussion

### Genome-Wide Identification of CIPK Gene Family in Pear

**FIGURE 1:**
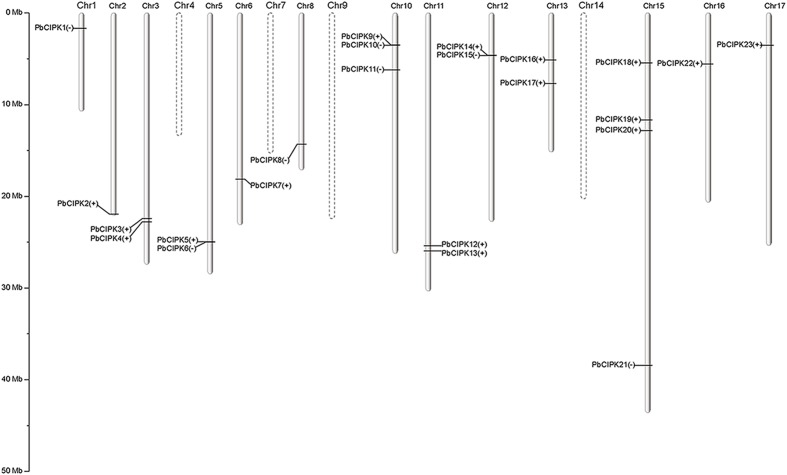
**Distribution of *PbCIPK* genes on pear chromosomes.** The roman numerals on top of each chromosome represent the number of the chromosome. The plus or minus accompanied with each *PbCIPK* gene indicates the stand direction of gene encoding. The dashed line of chromosome represents no distribution of *PbCIPK* gene.

Calcineurin B-like interacting protein kinases are evolutionarily conserved (Luan, 2009). Most flowering plants typically encodes around 30 CIPK homologs that harbor a protein domain in N-terminal and a NAF domain in C-terminal (Yu et al., 2014; Manik et al., 2015). In previous reports, 26 CIPK member in *Arabidopsis* (Weinl and Kudla, 2009), 30 in rice (Xiang et al., 2007), 27 in poplar (Yu et al., 2007), 23 in canola (Zhang et al., 2014), and 25 in cassava (Hu et al., 2015) were characterized. Most *CIPK* genes in higher plants have been reported in response to complex environmental conditions (Xiang et al., 2007; Chaves-Sanjuan et al., 2014; Tang et al., 2015). In this report, we identified putative *CIPK* genes in pear genome (Wu et al., 2013). Thirty *CIPK* genes were predicted to contain both the Pkinase-domain and NAF domain, and 33 protein sequences were found based on CIPK protein sequences from *Arabidopsis* and rice using BLASTP program with identity >50% (Supplementary Table S2). Finally, 28 gene models were overlapped by two separate searches and considered as putative *CIPK* genes (Supplementary Table S2). Their corresponding protein sequences, coding sequences and genomic sequences were collected from the pear genome for further analysis. The 28 genes of *CIPK*s from the pear genome are uniformly designated as *PbCIPK* followed by an Arabic number 1–28 according to the position of their corresponding genes on chromosomes 1–17 (**Figure 1**) and in the order of the scaffolds (**Table 1**). Subsequently, the related characteristics and multiple sequence alignments of the 28 putative *PbCIPK* genes in this study were analyzed (**Supplementary Figures S1** and **S2**; **Table 1**). All of the identified CIPK proteins from pear and *Arabidopsis* were aligned and most harbored similarly conserved architectures (**Supplementary Figures S1** and **S2**). Each PbCIPK protein contained both Pkinase domain and NAF domain, which is a core characteristics of CIPK (Luan, 2009; Chaves-Sanjuan et al., 2014), except that PbCIPK8, which has an incomplete Pkinase domain in its N-terminal (**Supplementary Figure S1**). The coding sequence length of CIPKs ranged from 789 to 1491 bp, and their encoded proteins varied from 262 to 496 amino acids in length. Their corresponding molecular weight (MW) varied from 48.73 to 50.99 kDa with isoelectric point (pI) values from 4.84 to 9.70 (**Table 1**). In addition, orthologous analysis of *CIPK*s across *P. bretschneideri* and *A. thaliana* indicated that 28 PbCIPK were linked to their corresponding 13 orthologs in *Arabidopsis* (**Table 1**), which suggested that gene duplications events occurred in the pear *CIPK* family.

### Genomic Location, Organization, and Phylogeny of PbCIPK Gene Family

The *PbCIPK* genes were mapped onto all chromosomes except chromosomes 4, 7, 9, and 14 in the pear (**Figure 1**). Twenty-three of 28 *CIPK* genes, were distributed unevenly on 13 of 17 chromosomes in pear, and each of these chromosomes contained at least one *PbCIPK* gene (**Figure 1**). Chromosome 15 contained four, or ∼18%, the greatest number, of CIPK family genes (**Figure 1**). The remaining five *PbCIPK* genes were also located on three scaffolds (scaffold235.0, scaffold958.0.1, and scaffold1158.0; **Table 1**). Scaffold235.0 and scaffold958.0.1 both contained two CIPK genes, corresponding to *PbCIPK24*, *-25* and *PbCIPK26*, *-27*, whereas scaffold1158.0 only contained one CIPK gene (*PbCIPK28*; **Table 1**).

**FIGURE 2:**
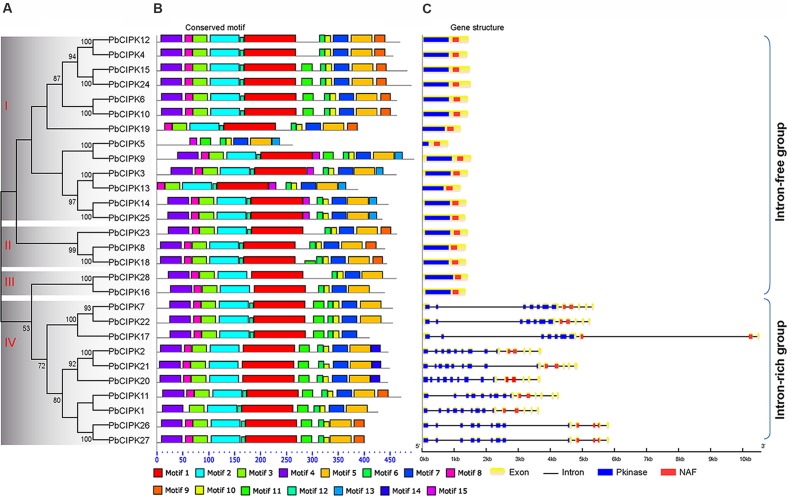
**Phylogenetic and structural analysis of PbCIPK family.** Phylogenetic tree of the PbCIPK family in scientific name of pear was generated using neighbor-joining with 1000 bootstrap in MEGA 6.0. Motif analysis was performed by MEME software (http://meme-suite.org/tools/meme), and gene structure was detected by GSDS 2.0 ((http://gsds.cbi.pku.edu.cn/).

In order to classify the CIPK family in pear, full-length protein sequences of all identified PbCIPKs were used to construct phylogenetic trees. Dues to the tree topologies produced by ML and NJ methods have largely consistent, the NJ phylogenetic tree was selected to show in **Figure 2A**. The 28 PbCIPKs were divided into four groups I, II, III, and IV on the basis of their phylogeny, gene structure, and conserved motif distributions (**Figure 2**). Simultaneously, we compared the phylogenetic groups and gene structures among *Arabidopsis* and pear (**Supplementary Figure S3**), and PbCIPKs had similar distributions in the phylogenetic clades and intron–exon structures of these genes in both species. Orthologous genes from *Arabidopsis* and pear were closely converged in the same subclusters (**Supplementary Figure S3**), which supported the organization of CIPK family in pear. Among four phylogenetic groups in pear, Group I and IV contained more genes than other groups. For example, group I included *PbCIPK3*, *-4*, *-5*, *-6*, *-9*, *-10*, *-12*, *-13*, *-14*, *-15*, *-19*, *-24*, *-25*, whereas Group IV included *PbCIPK1*, *-2*, *-7*, *-11*, *-17*, *-20*, *-21*, *-22*, *-26*, *-27*. The *PbCIPK*s classified in Groups III and IV were consistent with previous reports of the phylogeny of CIPKs contained in clades III and IV from *Arabidopsis*, Populus, and cassava (Yu et al., 2007; Hu et al., 2015).

Based on full protein sequences of CIPKs, we also investigated the phylogenetic relationship between pear and other species (*Arabidopsis*, rice, *B. distachyon*, soybean, peach, poplar, and grape; **Supplementary Figure S4**). All of the CIPKs from eight species grouped into the same four distinct groups (I to IV), which is consistent with the organization of CIPK family in pear (**Supplementary Figure S4**). This is in agreement with the current understanding of plant CIPKs expanded within four subgroups along with evolutionary relationships among these organisms (Adams and Wendel, 2005; Kleist et al., 2014; Zhu et al., 2016). Among eight plant species, the phylogenetic reconstruction of CIPKs further supported that the expansion patterns of pear CIPK gene family originated from groups I and IV in its evolutionary history, implying that they derived from duplications that occurred during recent genome duplication. In addition, the CIPKs from different species that have sequence similarity or conservation suggest that they could have similar functions in adaption or evolution.

### Gene Structure and Conserved Motif Analysis of *PbCIPK* Genes

The conserved motifs and intron–exon distribution of *PbCIPK*s were analyzed to better understand the structural features of pear *CIPKs* (**Figures 2B,C**). The conserved motif analysis of PbCIPKs supported the indicated phylogenetic relationship and classification of pear CIPKs (**Figures 2A,B**). Fifteen conserved motifs in pear CIPKs were detected and all pear CIPK proteins contained motifs 6 and 10 (**Figure 2C**; Supplementary Table S3). Motifs 1, 2, 3, and 4 were annotated as Pkinase domain (Supplementary Table S3). Motif 6 and 10 that are closely related to NAF domain (Pfam no. PF03822). Motif 6 contained the core NAF residues that are similar to the NAF domain in the PbCIPK family (**Figure 2C**; Supplementary Table S3). The motif distribution demonstrated the conserved structure of PbCIPK family. Among 28 PbCIPK proteins, almost all PbCIPK, except PbCIPK5, proteins contained the complete protein kinase and NAF domains, and the NAF domain. PbCIPK5 has an incomplete kinase domain (**Supplementary Figure S1**), but has the complete regulatory domain, the N-terminal, and NAF domain that is harbored in motifs 6 and 10. In additional, the conserved NAF domain is the key motif component to mediate CBL-CIPK physical interactions (Kolukisaoglu et al., 2004; Luan, 2009; Weinl and Kudla, 2009; Yu et al., 2014; Manik et al., 2015; Tang et al., 2015). The formation of stable CIPK and CBL complexes is necessary to regulate various ion transporters and abiotic stress responses in plants (Albrecht et al., 2001; Kolukisaoglu et al., 2004; Luan, 2009; Chaves-Sanjuan et al., 2014). Moreover, PbCIPKs had similar distributions of exons and architecture of the conserved domains within the same group. The phylogenetic relationship and the classification of PbCIPKs is supported by similarities in structures, and suggests that the roles of CIPKs with the characteristic conserved Pkinase and NAF domains genes in pear are involved in CBL-CIPK networks.

Gene structure analysis of *PbCIPK*s supported the phylogenetic groups and conserved motif distributions of PbCIPK family. The similar conserved motif distributions existed among members of *PbCIPK*s in the same phylogenetic group (**Figures 2A,C**). Intron–exon organization of the 28 *PbCIPK* genes indicated that *PbCIPK* genes could be classified into two clusters. The exon-rich cluster included only phylogenetic group IV (contained *PbCIPK1*, *-7*, *-11*, *-17*, *-20*, *-21*, *-22*, *-26*, and *-27*) with each gene containing 10–14 exons, and the exon-free subgroup included 19 genes in phylogenetic groups I, II, and III with no introns for most of them (**Figure 2C**). The conserved Pkinase and NAF domains, were interrupted by multiple introns at similar positions for the intron-rich cluster (**Figure 2C**), whereas members from groups I, II, and III in intron-free cluster were only had one complete exon. The similar intron rich/poor pattern of CIPK family members was also observed in both monocots and dicots species, such as *Arabidopsis*, rice, poplar, soybean, canola, and cassava (Kolukisaoglu et al., 2004; Yu et al., 2014; Zhang et al., 2014; Hu et al., 2015; Zhu et al., 2016). Because of high intron gain rates in earlier eukaryotic evolution, reduction of intron number in recent eukaryotic evolution is a common feature (Roy and Penny, 2007). Additionally, comparing with the intron distributions of *PbCIPK*s within different groups further reveals that the derivative forms of *PbCIPK*s are associated with the products of gene duplications, which are based on intron losses from the earlier multiple intron homologs. Thus, the expansion of CIPK family is dependent on the intron losses from the ancient multiple intron CIPK lineages along gene duplication.

### Gene Duplications and Divergence of *PbCIPK* Gene Family

**FIGURE 3:**
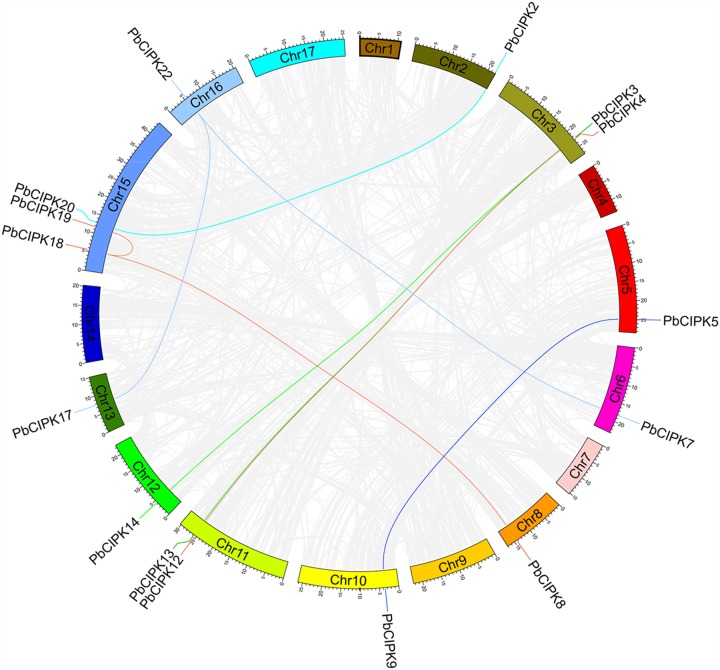
**Colinearity analysis *CIPK* gene family in pear genome.** All the pear *CIPK* family duplicated gene pairs are depicted in the pear chromosomes (Chr1-17). Gray background lines indicate collinear blocks in whole pear genome, and the collinear relationship of *PbCIPK* genes are indicated by solid color lines.

To investigate gene duplication of the *CIPK* gene family in pear, all possible paralogous gene pairs in all protein sequences of gene models (41019) of *P. bretschneideri* were searched. Blast hits and gene locations were used as the inputs for MCScanX (Wang Y. et al., 2012) to analyze all potential duplication events in the pear genome and to identify paralogous duplicated pairs of PbCIPK family (**Table 2**). Among *PbCIPK* family genes, nine duplicated pairs included 15 *PbCIPK* genes (*PbCIPK2*, *-3*, *-5*, *-7*, *-8*, *-12*, *-18*, *-22*, *-20*, *-13*, *-14*, *-9*, *-4*, *-19*, and *-17*) in **Table 2** with synteny (**Figure 3**). The synteny relationships of *PbCIPK* duplicated pairs were collected and visualized in **Figure 3** using Circos (Krzywinski et al., 2009). All duplicated events of *PbCIPK* gene pairs were derived from segmental duplication. The duplicated gene pairs mainly congregated on 11 chromosomes (2, 3, 5, 7, 8, 10, 11, 12, 13, 15, and 16). Moreover, the selection types and divergence dates of duplicated genes were investigated by calculating the synonymous (*K*s) and non-synonymous substitutions (*K*a) per site between duplicated pairs. *K*a/*K*s = 1 indicates neutral selection, *K*a/*K*s < 1 indicates purifying selection, and *K*a/*K*s > 1 indicates accelerated evolution with positive selection (Yang and Bielawski, 2000; Zhang et al., 2006). The *K*s, *K*a, and *K*a/*K*s of 11 duplicated gene pairs of *PbCIPK*s (**Table 2**) showed that all of the segmentally duplicated gene pairs in the *PbCIPK* family, whose *K*s ranged 0.007–1.233, and the *K*a/*K*s ratios of nine *PbCIPK* duplicated gene pairs ranged 0.142–0.292, suggesting that all duplicated genes of *PbCIPK* family had undergone purifying selection on the whole genome duplication (WGD). The recent WGD event in pear probably occurred 30–45 MYA (Velasco et al., 2010;Wu et al., 2013), whereas the divergence of pear and apple must have occurred 5.4–21.5 MYA (Wu et al., 2013). The divergence dates of the duplicated *PbCIPK* genes were also calculated, and ranged from 0.36 to 66.57 MYA (**Table 2**). The duplicated events of the *CIPK* family genes in pear is associated with the whole processes of the pear WGD, and the differentiation of apple and pear. Furthermore, the duplicated events were not synchronous in pear, and implicated their occurrences could be accompanied with the diverse functional differentiation to respond environments.

### *Cis*-Regulatory Elements Analysis of *PbCIPK* Genes

**FIGURE 4:**
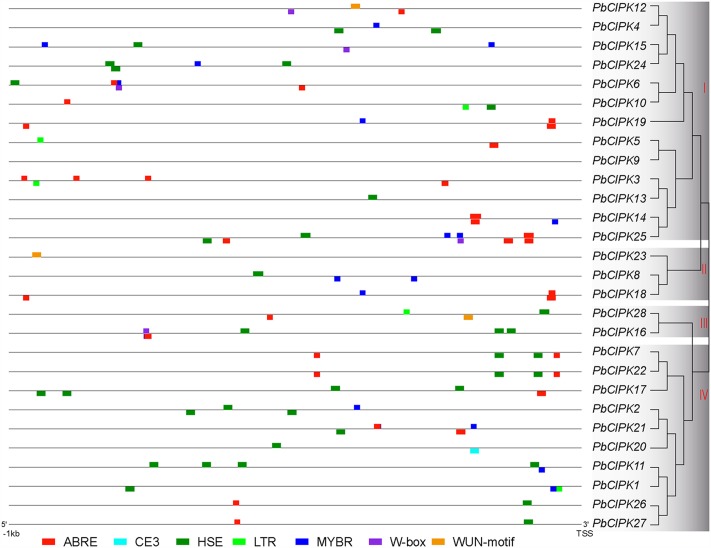
**Putative *cis*-elements in the promoter regions of *PbCIPK* genes.** Stress-responsive *cis*-elements ABRE (involved in the abscisic acid responsiveness), CE3 (involved in ABA and VP1 responsiveness), HSE (involved in heat and oxidative stress responsiveness), LTR (involved in the low-temperature responsiveness), MBR (MYB binding site involved in ABA and drought responsiveness), W-box (WRKY binding site involved in abiotic stress and defense response) and WUN-motif (involved in the wound responsiveness) were shown in the promoters regions (1000 bp upstream) of *PbCIPK*s.

The *cis*-regulatory elements, such as ABRE (ABA-responsive element), HSE (heat shock element), LTRE (Low-temperature-responsive element), MYBR (MYB transcription factor binding site), and W-box (WRKY transcription factor binding site) have been extensively characterized for their important roles under stress conditions (Yamaguchi-Shinozaki and Shinozaki, 2005; Xiang et al., 2007; Kim et al., 2011; Hernandez-Garcia and Finer, 2014). Stress-responsive elements were investigated in the promoter regions of *PbCIPK*s to explore the possible responsive mechanisms of pear CIPK genes to abiotic stress and detected in the 28 *CIPK* gene promoter regions (1000 bp upstream of the translation start site) of *PbCIPK*s. In the promoter regions of 27 *PbCIPK*s (except *PbCIPK9*), 110 *cis*-elements were involved in responding abiotic stresses (**Figure 4**). The number of stress-responsive elements in the selected promoter regions of 27 *PbCIPKs* ranged from the maximum in *PbCIPK25* (which contained 11 *cis*-elements) to the minimum in *PbCIPK23 and PbCIPK13* (which contained one *cis*-elements). Sixty-three percent (17/27) of the promoter regions of *PbCIPK*s contained ABRE elements and 71% (20/27) contained HSE elements (Supplementary Table S4). A LTR element was found in five promoter regions of *PbCIPK1*,-*3*,-*5*, -*10*, and -*28*. Eighteen MYBR elements and five W-box elements were located in the promoter regions of 14 genes and five genes, respectively. Moreover, other stress-responsive *cis*-elements were detected, including WUN-motif (Ni et al., 1996) in three genes (*PbCIPK12, PbCIPK23*, and *PbCIPK28*), and CE3 element (Shen et al., 1996) in one gene (*PbCIPK20*), respectively. Therefore, most *CIPK* family members in pear could be induced and transcriptionally regulated during different abiotic stresses.

### Expression Patterns of *PbCIPK*s in Response to Salt and Osmotic Stresses

**FIGURE 5:**
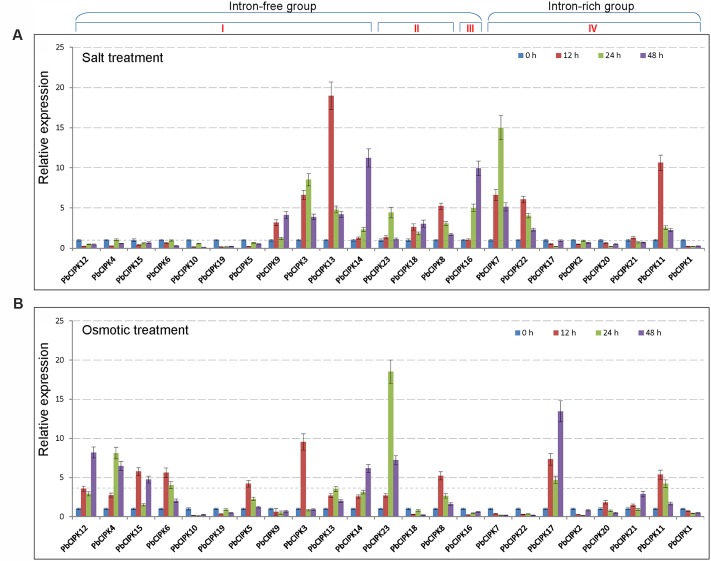
**Expression profiles of *PbCIPK* genes under salt and osmotic stresses.** Shoots with young leaves were exposed to 1/2 MS solution containing 200 mM NaCl or 15% (w/v) polyethylene glycol (PEG6000) for salt treatment **(A)** or osmotic treatment **(B)**, and leaves were sampled at 0, 12, 24 and 48-h for RT-qPCR analysis. The relative expression levels of 23 *PbCIPK*s in pear leaves were quantitatively calculated via the 2^-ΔΔCT^ method.

Calcineurin B-like interacting protein kinase family genes have been characterized to play an important role in plant response to salt or osmotic stress (Xiang et al., 2007; Weinl and Kudla, 2009; Chaves-Sanjuan et al., 2014; Manik et al., 2015; Pandey et al., 2015). To understand the responsive patterns of *PbCIPK* genes to salt and osmotic stresses, we selected all 23 *PbCIPK* genes, which can be mapped onto the pear chromosomes for RT-qPCR analyses (**Figure 5**). For salt treatments, among the 23 *PbCIPK*s, 12 genes (*PbCIPK3*, -*7*, -*8*, -*9*, -*11*, -*14*, -*16*, -*18*, -*21*, -*22*, and -*23*) were up-regulated, and 11 genes (*PbCIPK2*, -*4*, -*5*, -*6*,-*10*, -*12*, -*15*, -*17*, -*19*, and -*20*) were down-regulated (**Figure 5A**). For osmotic treatments, among 23 genes, 14 genes (*PbCIPK3*, -*4*, -*5*, -*6*, -*8*, -*12*, -*13*, -*14*, -*15*, -*17*, -*20*, -*21*, and -*23*) were up-regulated, and the remaining nine genes (*PbCIPK1*, -*2*, -*7*, -*9*,-*10*, -*16*, -*18*, -*19*, and -*22*) were down-regulated under osmotic stress (**Figure 5B**). Most *PbCIPK* genes that simultaneously responded to two stresses (**Figures 5A,B**) contained similar components of stress-responsive *cis*-elements, and each gene had more than one ABRE *cis*-elements in the promoter region (**Figure 4**). *PbCIPK*s contained putative stress-responsive *cis*-elements (i.e., ABRE, LTRE, MYBR, or W-box) in their promoter regions that were induced by salt or osmotic stresses. Among the 23 stress-inducible genes, five genes (*PbCIPK3*, -*8*, -*11*, -*13*, and -*14*) were up-regulatory in response to salt and osmotic stress, and four genes (*PbCIPK1*, -*2*, -*10*, and -*19*) were down-regulated expression by salt and osmotic stress. The expressions of duplicated *PbCIPK* gene pairs indicated the diverse expression of divergences under salt or osmotic stresses. For example, in gene pairs of *PbCIPK3-PbCIPK13*, *PbCIPK3-PbCIPK14*, *PbCIPK7-PbCIPK22*, *PbCIPK4-PbCIPK12*, the expression trends were consistent by salt and osmotic treatments, which implicated the duplicated genes in the co-expression phenomena with different expression patterns. Expressions of gene pairs, *PbCIPK3-PbCIPK13*, *PbCIPK3-PbCIPK14*, were up-regulated by salt or osmotic stress, whereas gene pairs of *PbCIPK7-PbCIPK22*, *PbCIPK4* -*PbCIPK12*, had different expressions under salt or osmotic stress. Expressions of gene pairs of *PbCIPK7-PbCIPK22* were both up-regulated by salt stress, and were both down-regulated by osmotic stress. In contrast, *PbCIPK4-PbCIPK12* were both down-regulated under salt stress, and were both up-regulated under osmotic stress (**Figures 5A,B**). The duplicated genes in the *PbCIPK* family that were involved in the responses to salt or osmotic stresses also emerged responsive divergences, and suggests that they may have some functional differentiation in long adaptation. In contrast, some duplicated genes of the family also had co-responsive patterns to multiple stresses (Tang et al., 2014). In addition, *PbCIPK* genes from intron-free or intron-rich clusters were induced by a specific salt or osmotic treatment, suggesting that stress responsive expressions of those genes were not correlated with their intron-exon structures (Xiang et al., 2007). To confirm that some of these stress-responsive genes exhibit potentially common or specific expression patterns, these genes were analyzed by gene co-expression analysis.

### Co-expression Networks of *PbCIPK*s under Salt and Osmotic Stresses

**FIGURE 6:**
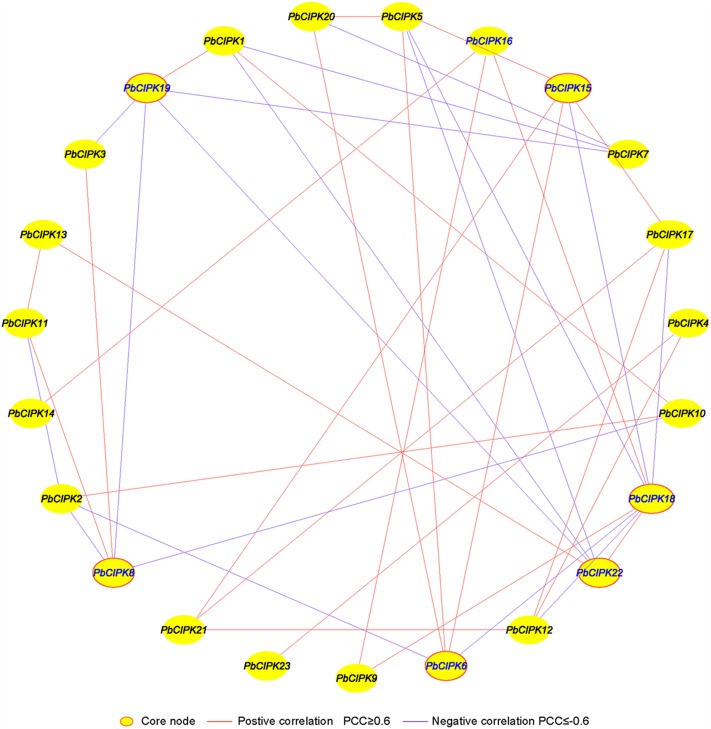
**Co-expression networks of *PbCIPK* genes under salt and osmotic stresses.** The co-expression networks were established based on the Pearson correlation coefficients of *PbCIPKs* gene pairs under salt and osmotic stresses, which involved 23 nodes and 108 regulatory edges. All of Pearson correlation coefficients of co-expression gene-pairs were significant at the 0.05 significance level (*p*-value), and the different edge line types indicated different relevance levels of co-expression gene-pairs.

Co-expression analysis has been employed to identify and discover some novel regulators or unknown mutual relationships by measuring large numbers of gene expressions involved in similar expression patterns under different conditions (Usadel et al., 2009; Movahedi et al., 2012; Bordych et al., 2013; Liang et al., 2014; Du et al., 2015). Previously, we utilized co-expression analyses tool to discover novel regulatory relationships and core nodes of networks (Tang et al., 2013). The gene co-expression networks of *PbCIPK*s were generated based on the Pearson correlation coefficients (PCCs) of stress-responsive *PbCIPK* genes under salt and osmotic treatments using RT-qPCR data. All PCCs that were significant at the 0.05 significance level (*p*-value) were collected and used to construct co-expression network by Cytoscape 3.3. A co-expression network of *PbCIPK*s mediating salt and osmotic stresses revealed 23 nodes and 39 regulatory edges (**Figure 6**). The 23 nodes representing 23 stress-responsive *PbCIPK*s were separately linked to each other through 39 edges representing PCCs between co-expression gene pairs (**Figure 6**; Supplementary Table S5). Among this network, 39 regulatory edges indicated 39 co-expression gene pairs of *PbCIPK*s that were linked with their PCCs. Each node harbored different number of regulatory edges, which varied from one to eight. Of the maximum number of regulatory edges, the *PbCIPK18* node contained eight regulatory edges, the second was enriched in the nodes *PbCIPK22*, *-19*, *-15*, *-8*, and *-6*, which all separately contained five regulatory edges (**Figure 6**). Co-expression of gene pairs (22/39 or 56%) had positive significant correlations, the remaining ∼44% co-expression gene pairs (17/39) had significantly negative correlations (**Figure 6**). The nodes of *PbCIPK22*, *-19*, *-18*, *-15*, *-8*, and *-6* could be placed in central roles in co-expression networks of *PbCIPK*s in response to abiotic stresses (**Figure 6**).

To validate the co-regulated relationships of *PbCIPK* genes, we constructed interaction co-regulatory networks of CIPK orthologs through retrieving STRING database^16^ based on the CIPK orthologs in *Arabidopsis*, which identified 13 high confidence interactive CIPK proteins (CIPK1,-3, -4, -7, -8, -9, -10, 11, -12, -14, -23, and -24) which were involved in stress-related regulatory networks (**Supplementary Figure S5**). In *Arabidopsis*, the CIPKs and CBLs interactions were involved in mediating different environmental stresses such as abscisic acid, cold, high pH, high salt, and osmotic stress (Xiang et al., 2007; Luan, 2009; Weinl and Kudla, 2009; Yu et al., 2014; Manik et al., 2015). *AtCIPK24/SOS2* can interact with AtCBL4/SOS3 to stimulate Na^+^/H^+^ exchange activity of the SOS1 to enhance salt stress tolerance in roots of *Arabidopsis* (Kudla et al., 2010; Manik et al., 2015). Among the 13 CIPK orthologs, each of them had at least three confidence interaction relationships, which targeted to CBLs (CBL1, -2, -4, -9, and -10) which were involved in calcium ion binding and related to sodium-hydrogen transport system. The co-regulatory relationships of *PbCIPK*s under salt and osmotic stresses were verified. Co-regulatory networks including *PbCIPK22*, *-19*, *-18*, *-15*, *-8*, and *-6* could be core mediators in the *PbCIPK*s response to salt and osmotic stresses. Although CIPKs mediate different abiotic stress and interact with different CBL proteins involved in multiple biological processes, there is still a need to identify possible response patterns and interaction networks of pear *CIPK*s for better understanding of their biological functions in responding to environmental stresses.

## Conclusion

In this study, we performed a comprehensive genome-wide survey of the CIPK family in pear. A total of 28 *CIPK* genes were identified and systematically analyzed for genomic locations, gene family organization, phylogenetic analyses, gene structure and conserved motif analysis, gene synteny and divergence timing, stress-responsive *cis*-element analysis, as well as gene expression profile and gene co-expression analyses under salt and osmotic stresses. Phylogenetic analyses of *PbCIPK*s indicated that 28 *CIPK*s from pear could be divided into four phylogenetic groups. Gene structure and motif analyses supported the classification of *PbCIPK*s, and each group shared similar or conserved distributions of exon-intron and protein motifs. Genomic location and synteny analyses indicated 23 out of 28 *PbCIPK*s were mapped on the pear chromosomes, nine gene pairs were derived from segmental duplications, and have maintained by purifying selections. Stress-responsive elements analysis indicated most promoter regions of *PbCIPK*s contained stress induced *cis*-acting elements, suggesting that these genes are involved in abiotic stress responses. Furthermore, expression profiles analysis of *PbCIPK*s under salt and osmotic stresses supported the prediction analysis of stress-responsive *cis*-elements in promoter regions. This indicated that almost all *PbCIPK*s could be induced by salt or osmotic treatment, and that some genes could be co-expressed in response to one or two stresses, which expressions exhibited similar or reverse patterns. Moreover, co-expression analyses indicated that 23 stress-responsive *PbCIPK*s had significant co-expression at 0.05 level under salt or osmotic stress, and some genes, such as *PbCIPK22*, *-19*, *-18*, *-15*, *-8*, and *-6*, acted as core regulators in response to salt and osmotic stresses. This study revealed the genome-wide identification of *PbCIPK* genes could be co-expressed under salt and osmotic stresses, and provide a basis for future research on abiotic stress responses medicated by CIPKs in pear.

## Author Contributions

YC, Z-MC, and JL conceived the study and designed the experiments. JT, JL, HL, XL, and QY performed the experiments and data analysis. JT wrote the manuscript. Z-MC and YC revised and proofread the manuscript. All authors read and approved the final manuscript.

## Conflict of Interest Statement

The authors declare that the research was conducted in the absence of any commercial or financial relationships that could be construed as a potential conflict of interest.

## Footnotes

1 http://peargenome.njau.edu.cn/

2 http://www.arabidopsis.org/

3 http://rice.plantbiology.msu.edu/

4 http://pfam.xfam.org/

5 http://hmmer.org/

6 http://smart.embl-heidelberg.de/

7 http://web.expasy.org/compute_pi/

8 http://meme-suite.org/tools/meme

9 http://prosite.expasy.org/

10 http://gsds.cbi.pku.edu.cn/

11 http://bioinformatics.psb.ugent.be/webtools/plantcare/html/

12 http://www.dna.affrc.go.jp/PLACE/

13 http://www.premierbiosoft.com

14 http://www.cytoscape.org/

15 http://string.embl.de/

16 http://string-db.org/

## Supplementary Material

**FIGURE S1 | Alignments of CIPK family proteins from pear (*P. bretschneideri*).**

**FIGURE S2 | Alignments of CIPK family proteins from pear (*P. bretschneideri*) and *A. thaliana.***

**FIGURE S3 | AML phylogenetic analysis of CIPK gene family in *P. bretschneideri* and *A. thaliana.***

**FIGURE S4 | ML phylogenetic analysis of CIPK gene family in eight plants.** Methods follow ML with 1000 bootstrap in MEGA 6.0 according to full length amino acid sequences from *A. thaliana*, *B. distachyon*, *G. max*, *O. sativa*, *P. richocarpa*, *P. persica*, *P. bretschneideri*, *V. vinifera*.

**FIGURE S5 | A confidence co-regulatory network of CIPK orthologs in *Arabidopsis*.**
