# Supplementary figures and images for: Characterization of CIPK Family in Asian Pear (Pyrus bretschneideri Rehd) and Co-expression Analysis Related to Salt and Osmotic Stress Responses
Source: Front Plant Sci. 2016 Sep 7;7:1361. doi: 10.3389/fpls.2016.01361 (PMC5013074; doi:10.3389/fpls.2016.01361)

Figure S1 Alignments of CIPK family proteins from pear (*P. bretschneideri*)

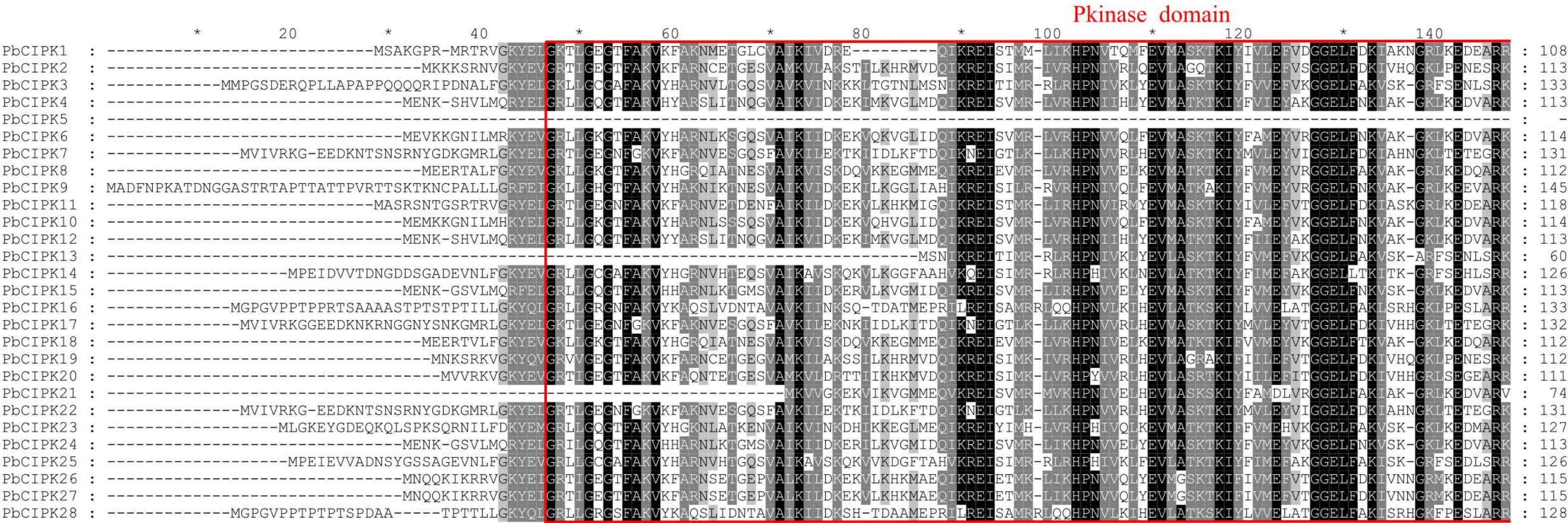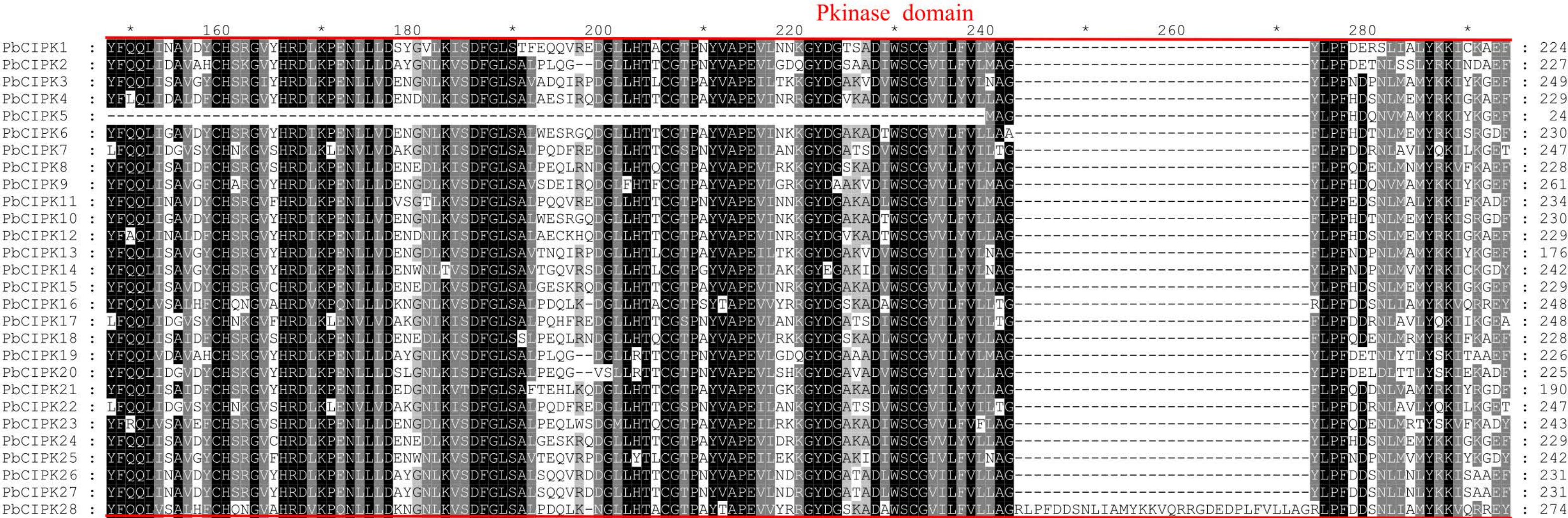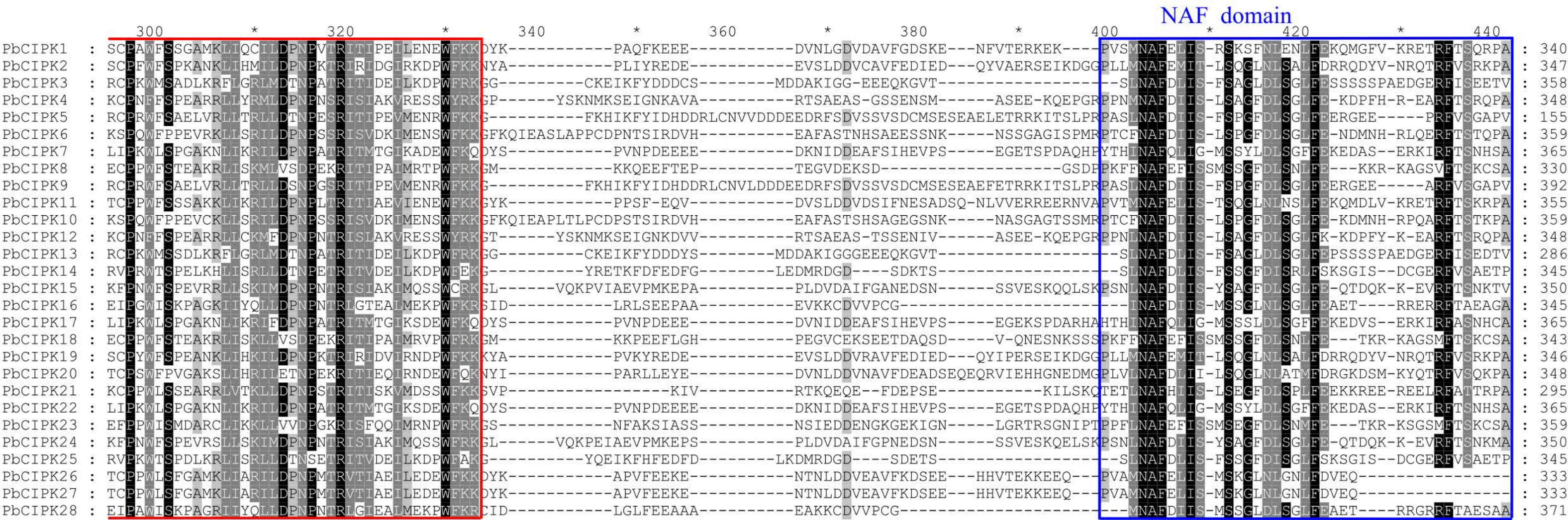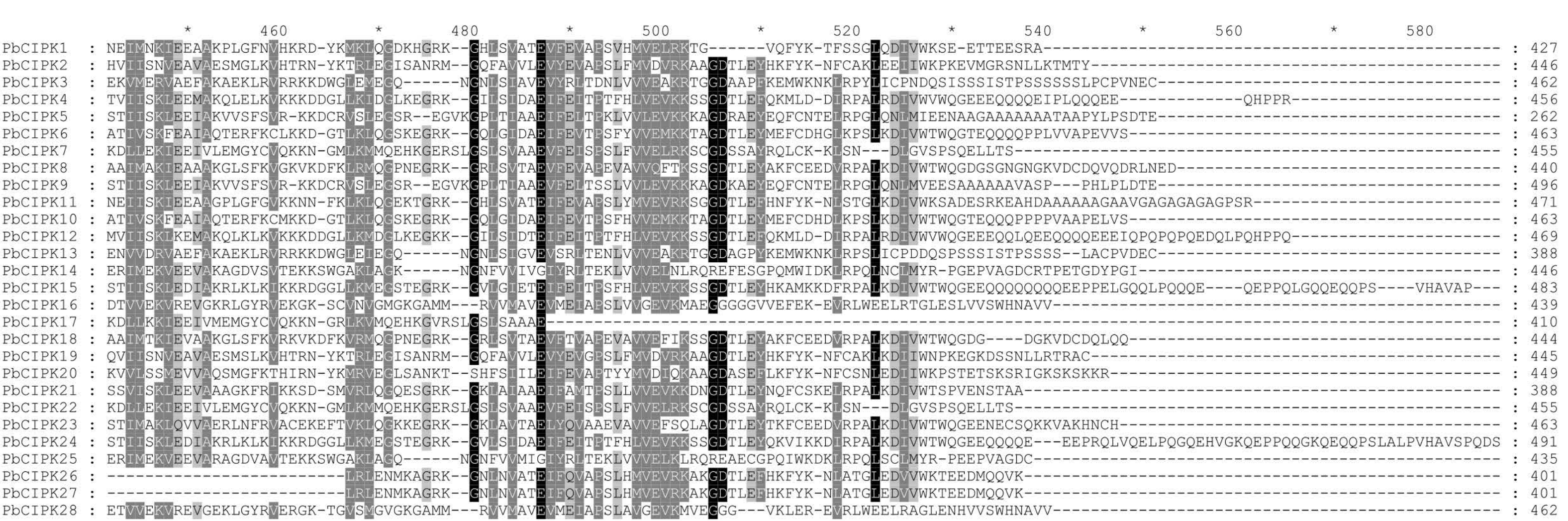

Supplement: Supplementary file 1 [file Image_1.PDF]

**Figure S2 Alignments of CIPK family proteins from pear (*P. bretschneideri*) and *A. thaliana***

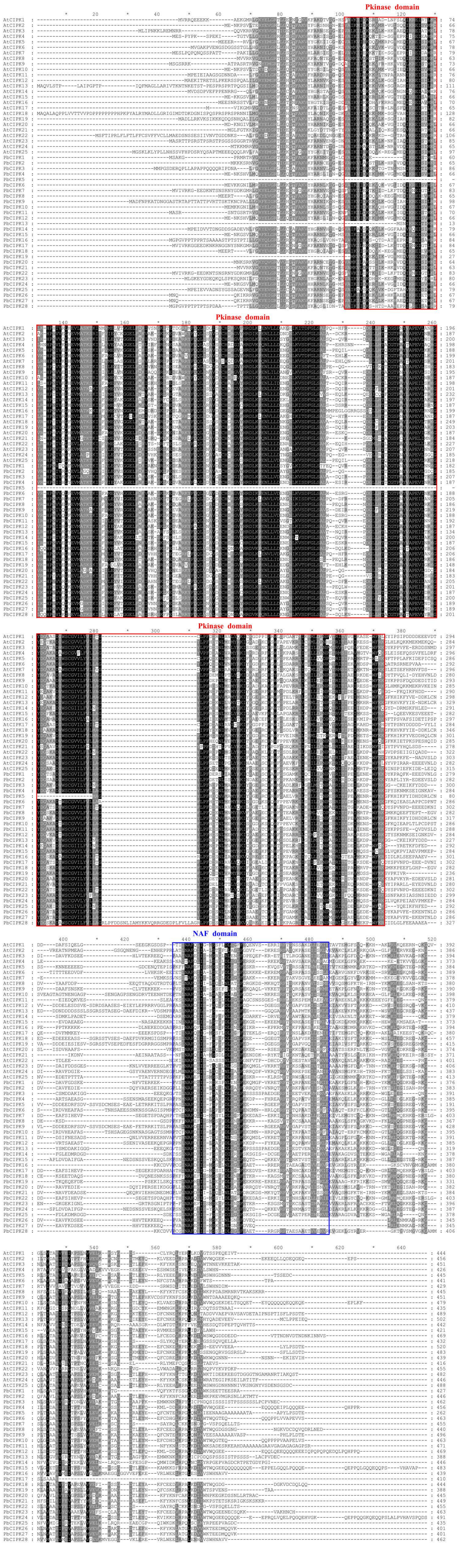

Supplement: Supplementary file 2 [file Image_2.PDF]

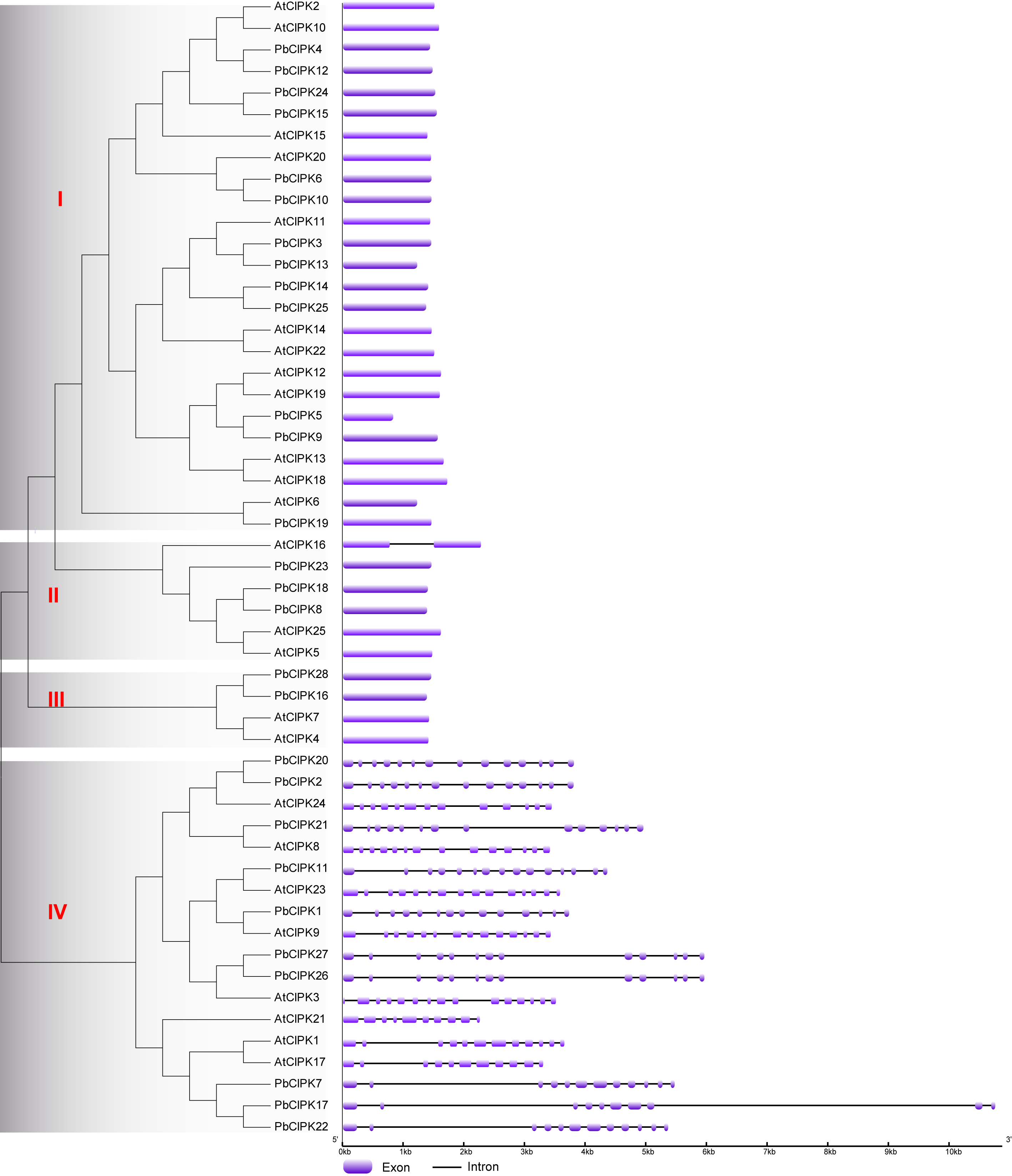

Supplement: Supplementary file 3 [file Image_3.TIF]

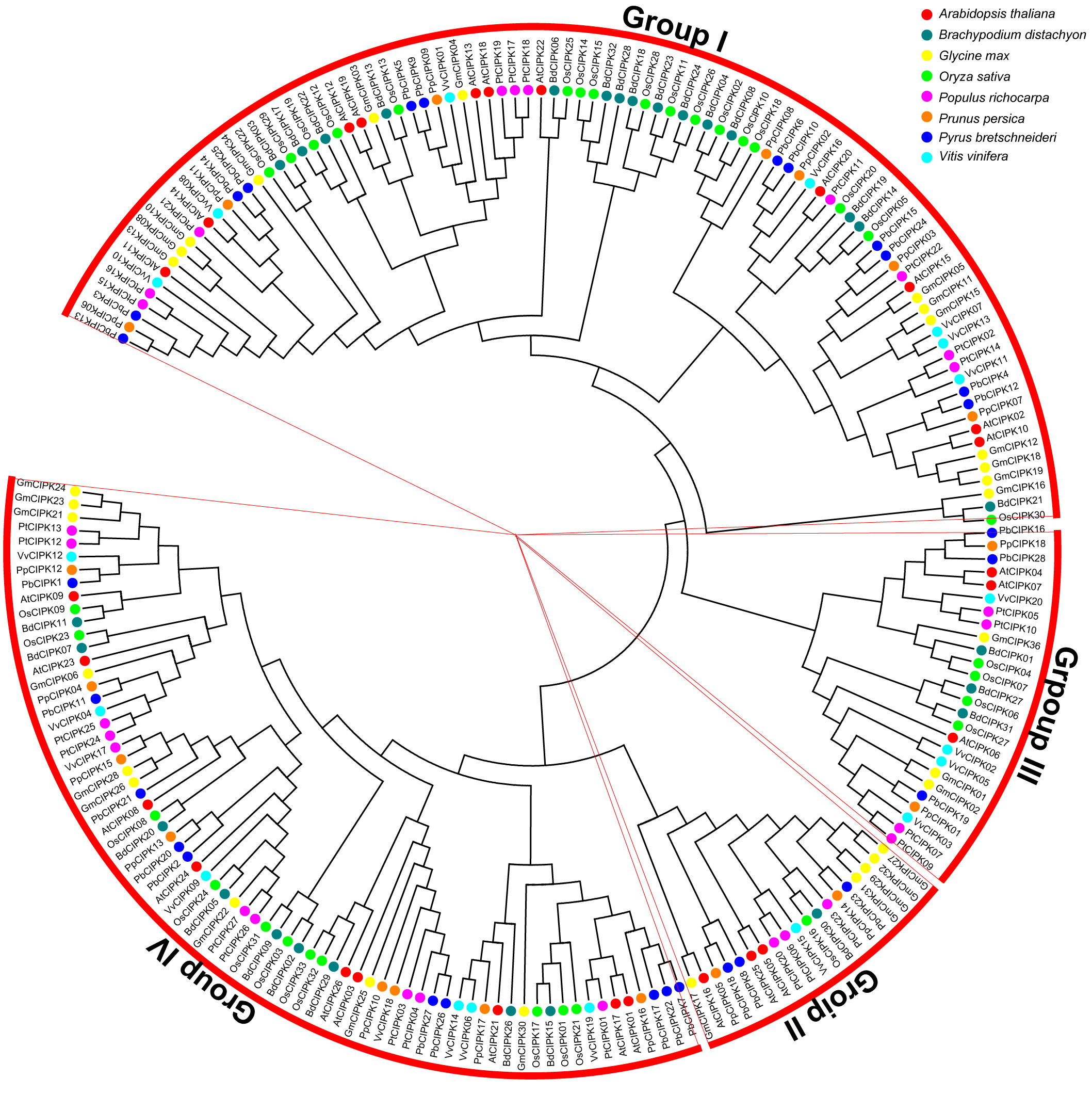

Supplement: Supplementary file 4 [file Image_4.TIF]

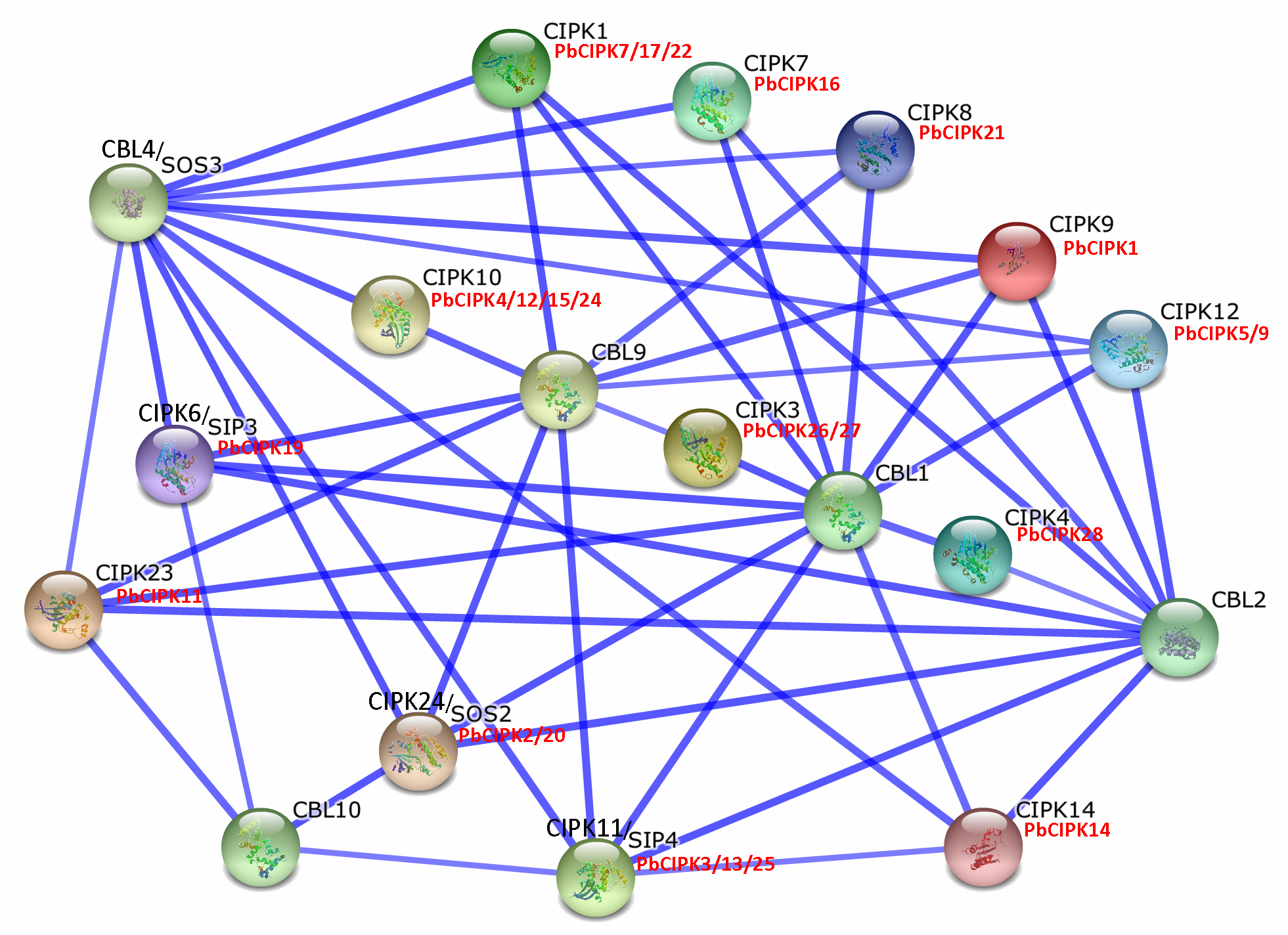

Supplement: Supplementary file 5 [file Image_5.TIF]
